# Association between beauty standards shaped by social media and body dysmorphia among Egyptian medical students

**DOI:** 10.1038/s41598-025-95617-3

**Published:** 2025-04-15

**Authors:** Mohammed N. Abdelaziz, Ahmed R. A. Moustafa, Hajer Azzam, Anwar M. Bshar, Ismail S. Ismail, Omnia Yousry Elhadidy

**Affiliations:** 1 Medical Intern, Faculty of medicine, Mansoura University, Mansoura, Egypt; 2 Integrated Medical Program, Mansoura University Faculty of Medicine, Mansoura, Egypt; 3 Assistant Lecturer of Psychiatry, Mansoura University, Mansoura, Egypt

**Keywords:** Body dysmorphia, Social media, Unattainable beauty, Medical students, Human behaviour, Psychology, Health care, Medical research

## Abstract

This study examines the relationship between exposure to unattainable beauty standards via social media and the prevalence of Body Dysmorphic Disorder among medical students in Egypt. The rapid development of digital platforms, particularly social media, has brought about a wider dissemination of unattainable beauty standards that may contribute to body image disorders and psychological problems. Given the unique pressures faced by medical students, who represent both consumers and influencers in health-related content, the current study attempts to ascertain whether excessive engagement with distorted beauty portrayals correlates with higher rates of BDD symptoms in this population. This was a cross-sectional questionnaire-based study consisting of 1126 undergraduate medical students, with a mean age of 20.8 years enrolled in any Egyptian medical school registered in the academic year 2023–2024, specifically from August–October 2024, except non-medical, graduate, and non-Egyptian students who met the exclusion criteria. We privately gathered answers via colleagues and electronically via online Google forms posted on social media groups. To our knowledge, this is the first study to investigate the relationship between social media use and BDD among medical students. According to social media practices, WhatsApp, Facebook, Instagram, YouTube, and TikTok were mostly used for 4–7 h daily. Most rarely or sometimes, take selfies, edit them with filters, and share them with others. The summary of BDDQ answers demonstrated that 6.3% of Egyptian medical students enrolled met the criteria for BDD. The majority reported that they do not like their face, and this leads to suffering from bullying in school or work, resulting in avoiding certain clothes as an avoidance behavior. The majority reported engaging in positive self-talk, participating in offline activities or hobbies, and unfollowing accounts promoting unattainable beauty standards as a coping strategy against unattainable beauty standards shaped by social media. Our study found that BDD is highly prevalent among social media users, especially on text-based platforms. The prevalence of BDD among Egyptian medical students is 6.3%, which is higher than worldwide. Interestingly, Egyptian medical students enrolled in our study believe that promoting body positivity, educating users about the risks of body dysmorphia, restricting content that promotes unrealistic body standards, and providing resources and support for those affected, respectively, are the critical measures that social media platforms should take to address body dysmorphia.

## Introduction

Body image refers to how an individual perceives, thinks about, and feels about their body. It encompasses how one visually represents their body, such as in a mirror reflection, and is influenced by societal constructs shaped by cultural and societal standards. This understanding is formed through body ideals heavily propagated by media, family, and peers^1^. Media platforms have had a major impact on Body image, as there is strong evidence suggesting the negative effects of objectifying beauty standards presented on mass media such as TV and Magazines^2^. For the past 2 decades, social media has replaced traditional media as the main outlet for communication and sharing cultural norms that influence today’s beauty standards. Facebook is considered the main priority social media app in Egypt, with 56 million active users and its greatest user demographic ranging from 18 to 44 years of age^3^. But how does social media affect body image? Social media allows individuals to upload idealized versions of themselves on their accounts; these images are often exaggerated with the use of filters and edits. Repeated exposure to these images leads to an increase in social comparison and internalization of these beauty ideals and thus leads to worsening dissatisfaction with body image. Body dissatisfaction has unfortunately been linked to psychological stress, which can result in a variety of psychological disorders, such as body dysmorphic disorder (BDD) and anorexia nervosa^4^.

BDD is an under-recognized but relatively common psychiatric disorder with a worldwide prevalence of 1.7 to 2.4%. A recent study conducted in Saudi Arabia has concluded that almost 9.5% of the patients visiting the dermatology clinic have BDD, which shows that there is an alarming lack of awareness about this disorder^5^. DSM V has categorized BDD as a disorder associated with a constant preoccupation of an individual’s perceived visual look, with repetitive behaviors aimed at hiding or fixing any possible flaws, although these flaws may be unobserved or minimally visible to others. Such behaviors must have a significant toll on the daily activities and social life of the patient and must not be associated with any other psychiatric disorder, i.e., eating disorders (Anorexia Nervosa, Bulimia Nervosa)^6^. According to various studies, BDD is most common in adolescents ranging between 13 and 18 years of age, and according to the CAPMAS (Central Agency for Public Mobilization and Statistics), the percentage of youth in EGYPT in 2021 statistics is 21 million, which forms about 21% of the population. Thus, this makes Egypt a significant breeding ground for individuals with BDD^7^. There has been no study discussing the association between BDD and social media usage among Egyptian medical students, the attitude that should be adopted to deal with its symptoms, and the role of social media platforms in amplifying its effects on their mental health. Thus, this study was conducted to evaluate the prevalence of body dysmorphic disorder (BDD) symptoms among Egyptian medical students, and the association between social media usage, and body dysmorphia.

## Methods and materials

### Study design and participants

This questionnaire-based cross-sectional study was conducted in Egypt. The study population targeted Egyptian medical students during August–October 2024. The participants were Egyptians of both genders, active on at least one social media platform, and not diagnosed with mental disorders. Non-social media users, non-Egyptians, and those with severe mental health conditions were excluded from the study as per the exclusion criteria. We informed participants at the beginning of the questionnaire that they were excluded if they had any mental disorders. So we excluded participants who self-reported having a mental health disorder because the study aimed to isolate the impact of social media on BDD in individuals without pre-existing mental health conditions that could significantly influence body image. The sample size was calculated online (https://www.openepi.com/SampleSize/SSPropor.htm). Assuming that the estimated proportion of the general population who met the criteria of BDD from a previous study in Saudi Arabia is 24.4%, according to Ateq Khadijah. et al.^8^ (p), with a 5% margin of error, and a 95% confidence interval, the minimum required sample size is 283. By adding a 40% expected drop rate, the final minimum sample size is 396. In our study, we used convenience sampling methods to recruit participants, which involved selecting medical students who were readily accessible and willing to participate. While we aimed to include students from various governorates to reflect geographic diversity, no formal weighting was applied to balance representation across regions. The survey was distributed through representatives from each governorate using an instant messaging app (What’s App, Facebook, messenger Inc.) and a direct link supplied by the survey administration software (Forms, Google Inc.). To overcome potential biases, we implemented strategies such as clear communication about the survey’s purpose and reminders to encourage participation. After answering all the questions, participants were instructed to send the web form to the web server. Later, the data was transmitted via the webserver to an Excel spreadsheet (version 16.0, Microsoft Corp., Albuquerque, NM, USA) for assessment.

### Data collection procedure

A structured questionnaire comprising four sections: demographic information, social media usage, body dysmorphic disorder concern questionnaire, and coping mechanisms and solutions, was developed based on a review of existing literature^8,9^ and was validated by a panel of experts in public health and psychiatry departments. Reliability and internal consistency were evaluated using the Cronbach alpha test. We conducted a pilot study on 32 students to test the reliability of the questionnaire. BDDQ (BDDQ) showed an accepted reliability value (Cronbach’s Alpha = 0.62). The questionnaire was administered in English as medical students are well-oriented with these concepts in the questionnaire. This questionnaire was designed with four consecutive sections to evaluate the link between body dysmorphic disorder and unattainable beauty standards shaped by social media. The first section of the questionnaire inquired about age, gender, academic level, socioeconomic situation, marital status, and residence. The second portion of the questionnaire analyzed social media use and behavior using two multiple-choice questions and three 4-point Likert questions, such as “To what extent do you take selfies?” (Always, Sometimes, Rarely, or Never). In addition, the following question was used to assess social media exposure: “How much time do you usually spend on social media?” (Low exposure is defined as less than 1 h per day, average exposure is 1–3 h per day, and high exposure is 4–7 h or more). The third part was the body dysmorphia disorder questionnaire. It was composed of five close-ended questions, each had a yes or no answer. The first and second questions asked the participants whether their appearance and their thoughts about it worried and preoccupied them. A yes answer to both of them was a must. The third question assessed the level of distress and interference with social and work life that these thoughts caused. It had 4 parts, a yes answer to any was a must. The fourth question asked about the average time spent per day thinking about appearance. Spending 1 h or more was a must to diagnose BDD. Thus, BDD was diagnosed on a total score of 4 or more. The fifth question asked about the fear of getting too fat. A yes answer to this question excluded the participants from BDD diagnosis due to the probability of confounding with eating disorders. A no-answer to question 5 was a must without an additional score. The fourth part examined coping mechanisms and solutions through two multiple choice questions and one 5-point Likert question (1 = strongly disagree; 5 = strongly agree), for example, “What strategies do you use to cope with negative feelings about your body image due to social media?” and “Would you support initiatives aimed at raising awareness about social media-related body dysmorphia?”

### Statistical analysis

Data were processed with IBM-SPSS software (IBM Corp., 2020). IBM SPSS Statistics for Windows, Version 27.0 (Armonk, NY: IBM Corporation). Categorical data were described with numbers and percentages. Continuous data were described using mean and standard deviation from normality charts. The chi-square test was used to compare frequencies. For multinomial categorical variables, dummy variables were created to indicate the effect of each category using chi-square. Multivariate binary logistic regression was conducted to identify predictors of BDD. Significant results were defined as a p-value < = 0.05.

## Results

Our sample consisted of 1126 undergraduate medical students, with a mean age of 20.8 years (1.6). 719 (63.9%) were females, and 407 (36.1%) were males. The majority were from Dakahlia, Cairo, Qena, Damietta, and Giza, 327 (29%), 282 (25%), 160 (14.2%), 82 (7.3%), and 45 (4%), respectively. The economic status of the majority, 1044 (92.7%), was middle level. The economic status was classified based on income brackets (low: < 10,000 EGP; middle: 10,000–30,000 EGP; high: > 30,000 EGP). 1114 (98.9%), we’re single. 793 (70.4%) were from urban residences (Table 1).

The platforms of WhatsApp, Facebook, Instagram, YouTube, and TikTok were mostly used, 729 (64.7%), 626 (55.6%), 601 (53.4%), 485 (43.1%), and 332 (29.5%), respectively. Most participants spend 4 to 7 h and 1 to 3 h daily on social media, 563 (50%) and 374 (33.2%), respectively. The majority rarely or sometimes take selfies, 484 (43%), 454 (40.3%), sometimes or never edit selfies with filters, 348 (30.9%), 336 (29.8%), and rarely or sometimes share them with others, 446 (39.6%), 363 (32.2%), respectively (Table 2).

**Table 1:** Characteristics of the study population.

		Mean	SD	*N*	%
Age		20.8	1.6		
Sex	Male			407	36.1%
	Female			719	63.9%
Governorate	Dakahlia			327	29%
	Cairo			282	25%
	Qena			160	14.2%
	Damietta			82	7.3%
	Qalyubia			38	3.4%
	Giza			45	4%
	Sharqia			29	2.6%
	Kafr El-Sheikh			26	2.3%
	Gharbia			21	1.9%
	Port Said			19	1.7%
	Ismailia			21	1.9%
	Assuit			21	1.9%
	Beni Sweif			15	1.3%
	Alexandria			8	0.7%
	Suez			8	0.7%
	Sini			7	0.6%
	Others			17	1.5%
Economic status	High			72	6.4%
	Middle			1044	92.7%
	Low			10	0.9%
Marital Status	Single			1114	98.9%
	Married			9	0.8%
	Divorced			1	0.1%
	Widowed			2	0.2%
Location	Urban (City)			793	70.4%
	Rural (Countryside)			333	29.6%

**Table 2:** Social media practices.

Question	Answers	*N*	%
Which social media platform do you use the most?*	Facebook	626	55.6%
	Whatsapp	729	64.7%
	Instagram	601	53.4%
	Twitter	203	18.0%
	Snapchat	190	16.9%
	Tik Tok	332	29.5%
	Youtube	485	43.1%
	Linked in	35	3.1%
	Telegram	39	3.5%
	Other platforms	18	1.6%
Average time spent on social media per day	More than 7 h	167	14.8%
	4–7 h	563	50%
	1–3 h	374	33.2%
	Less than 1 h	22	2%
To what extent do you take selfies?	Always	96	8.5%
	Sometimes	454	40.3%
	Rarely	484	43%
	Never	92	8.2%
To what extent do you use filters to edit selfies?	Always	121	10.7%
	Sometimes	348	30.9%
	Rarely	321	28.5%
	Never	336	29.8%
To what extent do you share selfies with others?	Always	69	6.1%
	Sometimes	363	32.2%
	Rarely	446	39.6%
	Never	248	22%

*Multiple response analysis.

Our primary outcome was to assess the prevalence of Body Dysmorphic Disorder (BDD) in medical students. Only 71 (6.3%) participants met the criteria for BDD. The summary of BDDQ answers is shown in Table 3. 621 (55.2%) were worried about their appearance. Of them, 55.9% thought about it a lot as a problem, mostly their faces (16.7%), their abdomen (9.7%), weight (6.1%), noses 51 (7.9%), thighs (4.5%), and all their body (4.3%). The appearance problem upset 36.5% of students and interfered with the social activities of 26.9%. 27.6% reported avoidance behaviors, mostly towards clothes (27.7%), socialization (20.9%), and taking and publishing photos (9.6%). 84.7% spent 1 h or more per day thinking about their appearance. 44.9% reported that their main concern was they were not thin enough or might get too fat and thus were excluded from the BDDQ scores calculation.

Our secondary outcome was to explore the effect of sociodemographics and social media practices on BDD, as shown in (Table 4). 63.4% were females with a mean age of 20.8 (1.7). The age mean differed significantly between BDD and non-BDD (*p* < 0.001). We found a significant difference between BDD and non-BDD according to the Governorate (*p* = 0.03), especially Dakahlia (*p* = 0.002) and Qena (*p* < 0.001). A significant difference was also found regarding high economic status (*p* = 0.03) marriage (*p* = 0.05), and residence (*p* = 0.03). Multivariate logistic regression revealed the predictors of BDD as shown in Table 5. Participants from the Governorates of Dakahlia were less likely to have BDD (OR = 0.34, *p* = 0.003, 95% CI 0.17–0.69). However, participants were more likely to have BDD if they were 21 years old or less (median age) (OR = 2.56, *p* = 0.004, 95% CI 1.36–4.82), from Qena (OR = 2.56, *p* = 0.001, 95% CI 1.48–4.43), high economic status level (OR = 2.29, *p* = 0.03, 95% CI 1.09–4.81), and rural residence (OR = 1.71, *p* = 0.03, 95% CI 1.04–2.79).

Participants reported the strategies they use to cope with negative feelings about their body image (Table 6). 445 (39.5%) engage in positive self-talk, 353 (31.3%) participate in offline activities or hobbies, and 343 (30.5%) unfollow accounts promoting unattainable beauty standards. Also, 331 (29.4%) limit social media usage, 269 (23.9%) seek support from friends or family, and 97 (8.6%) consult a mental health professional.

Participants suggested measures that social media should take to address body dysmorphia (Table 6). The majority suggested education about BDD risks 616 (54.7%), promotion of body positivity 536 (47.6%), and restriction of unfavorable content 510 (45.3%). They also suggested providing resources and support 484 (43%) and implementing mental health support features to 446 (39.6%). About half of participants, 550 (48.8%), strongly support initiatives aimed at raising awareness about social media-related body dysmorphia.

**Table 3:** Summary of body dysmorphic disorder questionnaire (BDDQ).

		*N*	%
1. Are you worried about how you look?	Yes	621	55.2%
	No	505	44.8%
2. If yes, Do you think your appearance problems a lot and wish you could think about them less? (*N* = 621)	Yes	347	55.9%
	No	274	44.1%
If yes, What are the body areas that you do not like?* (*N* = 621)	Face	104	16.7%
	Nose	49	7.9%
	Eyes	15	2.4%
	Hair	25	4%
	Teeth	24	3.9%
	Abdomen	60	9.7%
	Chest	21	3.4%
	Back	14	2.3%
	Shoulders	6	1%
	Arms	25	4%
	Hands	6	1%
	Nails	2	0.3%
	Hips	24	3.9%
	Thighs	28	4.5%
	Legs	12	1.9%
	Height	10	1.6%
	Weight	38	6.1%
	Skin	21	3.4%
	Shape	13	2.1%
	All	27	4.3%
3.			
a. Has this problem often upset you a lot?	Yes	411	36.5%
	No	715	63.5%
b. Has it often gotten in the way of doing things with friends, dating, your relationship with people, or your social activities?	Yes	303	26.9%
	No	823	73.1%
c. Has it caused you problems in School, work, or other activities?	Yes	131	11.6%
	No	995	88.4%
If yes, What are they?* (*N* = 131)	Avoid physical activities	9	6.9%
	Bullying	26	19.8%
	Going out with family and friends/Social anxiety	20	15.3%
	Low self-confidence/self-esteem	9	6.9%
	Feeling exhausted easily	5	3.8%
	Nervous/anxiety/feeling insecure	16	12.2%
	Depression	7	5.3%
	Studying	1	0.8%
	Prefer not to mention	54	41.2%
d. Are there things you avoid because of how you look?	Yes	311	27.6%
	No	815	72.4%
If yes, what are they?* (*N* = 311)	Clothes	86	27.7%
	Eating	11	3.5%
	Taking and publishing photos	30	9.6%
	Socializing/dating/going out with family/friends	65	20.9%
	Swimming/hobbies	11	3.5%
	Cosmetics/hairstyles/showing body	19	6.1%
	Prefer not to mention	122	39.2%
4. On an average day, how much time do you usually spend thinking about how you look?	>= 1 h	954	84.7%
	< 1 h	172	15.3%
5. Is your main concern with how you look that you aren’t thin enough or that you might get too fat?	Yes	506	44.9%
	No	620	55.1%

*Multiple response analysis.

**Table 4:** Associations between sociodemographics and social media practices and BDD.

	BDD (*N* = 71)				Non-BDD (*N* = 1055)				*p*-value
	Mean	SD	*N*	%	Mean	SD	*N*	%	
Age	20.8	1.7			20.4	1.3			< 0.001*
Gender									0.93
Male			26	36.6%			381	36.1%	
Female			45	63.4%			674	63.9%	
Governorate									0.03*
Dakahlia			9	12.7%			318	30.1%	0.002*
Cairo			24	33.8%			258	24.5%	0.08
Qena			20	28.2%			140	13.3%	< 0.001*
Damietta			4	5.6%			78	7.4%	0.58
Qalyubia			2	2.8%			36	3.4%	0.79
Giza			1	1.4%			44	4.2%	0.25
Sharqia			3	4.2%			26	2.5%	0.37
Kafr El-Sheikh			0	0.0%			26	2.5%	0.18
Gharbia			1	1.4%			20	1.9%	0.77
Port Said			2	2.8%			17	1.6%	0.45
Isamailia			0	0.0%			21	2.0%	0.23
Assuit			2	2.8%			19	1.8%	0.54
Beni Sweif			1	1.4%			14	1.3%	0.96
Alexandria			0	0.0%			8	0.8%	0.46
Suez			1	1.4%			7	0.7%	0.47
Sinai			0	0.0%			7	0.7%	0.49
Others			1	1.4%			16	1.5%	0.94
Economic status									0.06
High			9	12.7%			63	6.0%	0.03*
Middle			62	87.3%			982	93.1%	0.07
Low			0	0.0%			10	0.9%	0.41
Marital status									0.25
Single			69	97.2%			1045	99.1%	0.14
Married			2	2.8%			7	0.7%	0.05*
Divorced			0	0.0%			1	0.1%	0.8
Widowed			0	0.0%			2	0.2%	0.71
Residence									0.03*
Urban			42	59.2%			751	71.2%	
Rural			29	40.8%			304	28.8%	
Which social media platform do you use the most?									
Facebook			46	64.8%			580	55.0%	0.11
WhatsApp			46	64.8%			683	64.7%	0.99
Instagram			37	52.1%			564	53.5%	0.83
Twitter			11	15.5%			192	18.2%	0.57
Snapchat			12	16.9%			178	16.9%	1.00
TikTok			21	29.6%			311	29.5%	0.99
YouTube			26	36.6%			459	43.5%	0.26
Linked in			2	2.8%			33	3.1%	0.88
Telegram			3	4.2%			36	3.4%	0.72
Other platforms			2	2.8%			16	1.5%	0.40
Average time spent on social media per day									0.67
> 7 h			33	46.5%			530	50.2%	0.54
4–7 h			23	32.4%			351	33.3%	0.88
1–3 h			14	19.7%			153	14.5%	0.23
< 1 h			1	1.4%			21	2.0%	0.73
To what extent do you take selfies?									1.00
Always			30	42.3%			454	43.0%	0.9
Sometimes			29	40.8%			425	40.3%	0.93
Rarely			6	8.5%			86	8.2%	0.93
Never			6	8.5%			90	8.5%	0.98
To what extent do you use filters to edit selfies?									0.47
Always			23	32.4%			325	30.8%	0.78
Sometimes			20	28.2%			301	28.5%	0.95
Rarely			17	23.9%			319	30.2%	0.26
Never			11	15.5%			110	10.4%	0.18
To what extent do you share selfies with others?									0.64
Always			31	43.7%			415	39.3%	0.47
Sometimes			23	32.4%			340	32.2%	0.98
Rarely			15	21.1%			233	22.1%	0.85
Never			2	2.8%			67	6.4%	0.23

Categorical data are described by number and % calculated by column.

*Significant.

**Table 5:** Significant predictors of BDD.

	OR	*P*-value.	95% CI
Age group			
<=21	2.56	0.004*	1.36–4.82
> 21	R	R	R
Governorate			
Dakahlia	0.34	0.003*	0.17–0.69
Other Governorates	R	R	R
Qena	2.56	0.001*	1.48–4.43
Other Governorates	R	R	R
Economic status			
High	2.29	0.03*	1.09–4.81
Middle/low	R	R	R
Residence			
Rural	1.71	0.03*	1.04–2.79
Urban	R	R	R

*Significant.

*OR* Odds Ratio, *CI* Confidence Interval, *R* Reference.

**Table 6:** Adaptive mechanisms and their attitudes.

		*N*	%
What strategies do you use to cope with negative feelings about your body image due to social media?*	Limiting social media usage	331	29.4%
	Unfollowing accounts that promote unrealistic body standards	343	30.5%
	Seeking support from friends/family	269	23.9%
	Consulting a mental health professional	97	8.6%
	Engaging in positive self-talk	445	39.5%
	Participating in offline activities/hobbies	353	31.3%
	Watching shows/podcasts	4	0.4%
	Prayer/Quran/Meditate/self-embracing	57	5.1%
	Health care/improvement	40	3.6%
	Nothing	72	6.4%
What measures do you think social media platforms should take to address body dysmorphia?*	Promoting body positivity	536	47.6%
	Banning photo editing apps	282	25.0%
	Implementing mental health support features	446	39.6%
	Educating users about the risks of body dysmorphia	616	54.7%
	Restricting content that promotes unrealistic body standards	510	45.3%
	Providing resources and support for those affected	484	43.0%
	Prevent bullying/body shaming	2	0.2%
	Religious teaching/self-embracing	12	1.1%
	Nothing	30	2.7%
Would you support initiatives aimed at raising awareness about social media-related body dysmorphia?	Strongly support	550	48.8%
	Support	385	34.2%
	Neutral	161	14.3%
	Oppose	17	1.5%
	Strongly oppose	13	1.2%

*Multiple response analysis.

## Discussion

Body dysmorphic disorder is a common and often severe psychological condition. Given the high prevalence of social media use and BDD in young people, the current cross-sectional study sought to contribute to the small but growing body of research on the association between social media use and body dysmorphic symptoms. To the best of our knowledge, this is the first study to look at the link between social media use and BDD among Egyptian medical students. Our sample of 1126 undergraduate medical students had an average age of 20.8 years. The study revealed that 6.3% of individuals had BDD, significantly higher than the global norm of 2.2% in adolescents, 1.9% in adults, and 3.3% in students^10^. However, this finding aligns more closely with findings from non-Western regions, such as Saudi Arabia (8.8% in Jeddah) and Benin (10.31% at the University of Parakou)^11,12^, it contrasts with lower rates reported in Asian contexts. For example, a study of Chinese medical students found a BDD prevalence of 1.3%^13^, while Western epidemiologic data suggest general population rates of 1.7–2.9%^12,14^.

This disparity may reflect methodological differences (e.g., diagnostic tools, sampling strategies) or sociocultural factors. In Asian medical schools, academic pressures are intense, yet BDD rates remain relatively low compared to Egypt and Africa. This could stem from cultural variations in beauty standards—for instance, East Asian cultures often emphasize facial features or skin tone^13^, while Middle Eastern and African contexts may prioritize body shape or hair^11,12^. Additionally, social media’s role in amplifying appearance ideals appears more pronounced in regions with rapidly digitizing populations, such as Egypt and Saudi Arabia, where platforms like Instagram and Snapchat are widely used for image-centric interactions. Western studies of medical students are limited, but general population data suggest BDD prevalence is lower than in our sample. However, Western medical students face comparable academic stressors, which are known to exacerbate body image concerns^13^. The higher prevalence in our study and similar non-Western settings may also reflect underdiagnoses in Western contexts due to greater mental health literacy or differences in help-seeking behavior^12,14^.

For instance, the complex interactions of cultural, social, and psychological factors unique to the Egyptian context can explain this high percentage. Physical appearance is greatly prized in Egyptian society, particularly in terms of marriageability and social status. The 2023 Alexandria University study confirmed that 36.6% of students fixated on skin imperfections and 36.2% on belly size—traits subject to extreme scrutiny within Egyptian cultural standards^15^. This anxiety is fueled by traditional beauty standards that identify physical beauty and moral virtue, a subject examined in a 2022 study of Egyptian Muslim women^16^. Participants reported intense pressure to conform to limiting beauty standards, as deviation risks social exclusion or reduced marriage prospects^16^. In addition, the prevalence of image-sharing platforms like Instagram and TikTok has only served to exacerbate Egypt’s BDD symptoms. Researchers at Alexandria University confirmed a dose-response relationship: every additional hour per day spent on social media tripled the risk of BDD symptoms (OR = 2.926)^15^. Egyptian women are faced with conflicting pressures: with traditional modesty standards pitted against modern beauty standards as shown in media. The 2022 survey of plastic surgery discovered that 41% of Egyptian women presenting for cosmetic surgery cited “marriage market competitiveness” as a primary reason, many concomitant with female gender role stress (FGRS)^16^. Egypt’s BDD prevalence may be an artifact of underdiagnoses, not differences in actual incidence. This treatment avoidance persists: the 2023 Alexandria cohort illustrated that help-resistance tripled BDD risk (OR = 3.327)^16^.

In line with previous reports^10^, 63.4% of persons with BDD were female. Most medical students with BDD were single urban residents from Cairo, Qena, and Dakahlia governorates with a middle-level economic situation. Notably, social media platforms that rely mostly on text (like Facebook and WhatsApp) rather than images (like Instagram and YouTube) were highly linked to BDD. This result contradicts previous research^17–19^. This is because, in Egypt, text-based sites like WhatsApp are focal points of daily communication and social interaction. These sites provide opportunities for incessant appearance-related conversation—e.g., dieting, exercise, or appearance criticism—thus potentially increasing body dissatisfaction even without visual exposure. This is supported by^20^, which posits that the use of social media can impact mental health in different ways. Compared to the relatively passive social comparison which is typical of image-based websites, the explicit, personalized censure which is common in text-based communication could be more emotionally charged and trigger BDD-related issues^20^. Moreover, the academic pressure of medical students could uniquely combine with their utilization of social media. Given the intensity of their curriculum, medical students may have to rely heavily on text-based platforms like WhatsApp for organization and support in studying, thus possibly indirectly exacerbating stress and consequently BDD symptoms. Finally, our study involved platform frequencies of use, not types of content consumed. Our participants might have used image-based sites in more passive ways (e.g., watching educational videos on YouTube), and engaged actively in appearance discourses on text-based sites. People with BDD used social media on average for a lot longer than people without the disorder. Consistent with Alsaidan et al.^18^, the majority used social media platforms for more than 4 h each day. At the same time, Ateq et al.^8^ reported that the majority of participants spent < 4 h per day. Of the participants, 55.9% were incredibly concerned about their appearance. In the current study, the most worrisome body regions were the face (16.7%), belly (9.7%), and nose (7.9%). Concerns about abdominal appearance can be explained by the high prevalence of abdominal obesity and metabolic syndrome due to the socioeconomic, lifestyle, and nutritional changes that have been taking place in the Egyptian community and some other Arab countries toward the unhealthy pattern^21^. Cultural and social pressures can exacerbate facial concerns, especially in females, increasing the likelihood of developing BDD. Contrary to our study, these studies^18,22–24^ reported that skin and hair were the areas of highest concern. Only 11.6% of individuals claimed that their looks had caused problems in their community. In line with this Saudi study^18^, bullying and social anxiety were the most common issues at school or work, accounting for 2.4% and 1.8%, respectively.

Certain clothes, taking and publishing images, and socializing were the key acts avoided due to their appearance, resulting in various negative consequences: (1) Increased social withdrawal, (2) Reinforced negative self-image, (3) Obsessive checking and validation seeking, (4) Negative impact on academic performance, (5) Low self-esteem and professional confidence, (6) Increased risk of depression and anxiety. Social media can have varying effects on body image. Selfies, or frontal photos of oneself, can be taken with apps like Instagram and Snapchat and edited with a range of striking filters. To enhance one’s appearance, most filters alter facial features, usually in an exaggerated manner. People who have these traits may experience ongoing body dissatisfaction. Social media exposure to beauty standards exacerbates body image problems, per a recent comprehensive assessment of experimental investigations^25^. Additionally, a study^26^ carried out in psychiatric settings found that frequent selfie use has been connected to appearance concerns. Concerns about physical appearance increased as a result of redefining selfies, per another systematic review^27^. Another comprehensive analysis found that reframing selfies increased concerns about physical appearance^27^. The majority of respondents cited positive self-talk, offline hobbies and activities, unfollowing accounts that promote unrealistic body ideals, and restricting social media use as coping techniques for negative body image. When using photo editing apps, social media users pay greater attention to how they look, which promotes acceptance of fake beauty^28^. Frequent usage of filters and selfies can lead to an obsession with unrealistic beauty standards and a desire to alter one’s appearance.

## Limitations and recommendations

Limitations should be applied while evaluating the findings of this study. The study’s cross-sectional design precludes drawing any conclusions regarding the causal relationship between its variables. Nonetheless, experimental research has demonstrated a causal relationship between BDD and social media^29^. Our study’s second weakness is the convenience sampling strategy. The use of convenience sampling limits the generalizability of our findings, as certain governorates or groups may have been over- or under-represented. Thus, we recommend future studies employ probability-based sampling methods, such as stratified or random sampling, to ensure more balanced and representative samples. Since Facebook is the main communication tool utilized by the vast majority of men, women, and both younger and older populations in Egypt, we specifically employed it to reach all demographic groups. Furthermore, a far larger sample size than the suggested predicted power was used for the survey. It may be the goal of future research to include more guys in their sampling. Cross-sectional studies that use self-reported responses have an additional inherent constraint that could compromise response accuracy. Regarding the exclusion of non-social media users and the potential introduction of selection bias, this exclusion limits the generalizability of our findings to the broader population. We further suggest that future research explore BDD prevalence in both social media users and non-users to provide a more comprehensive understanding. This can be explained by the fact that our study specifically aims to assess the correlation between exposure to idealized beauty standards shaped by social media and body dysmorphic disorder (BDD) symptoms. Since social media is the primary source of the beauty standards under investigation, non-users would not have the same level of exposure. Thus, our findings are specific to social media users and may not be generalizable to the entire population. We excluded participants who self-reported having a mental health disorder because the study aimed to isolate the impact of social media on BDD in individuals without pre-existing mental health conditions that could significantly influence body image. We acknowledge that this exclusion significantly limits the generalizability of our findings and that our results only apply to individuals without self-reported mental health disorders. Thus, future research to investigate the relationship between social media, BDD, and mental health in a broader population is recommended. According to the limitations of our study population, our study was conducted among Egyptian medical students, a specialized group that may not be representative of the general population. The demanding academic environment could limit their social media use compared to other groups, potentially underestimating the impact of social media on body image and body dysmorphic disorder (BDD). Additionally, the influence of socioeconomic factors, age range, and cultural context on perceptions of beauty standards emphasizes that our findings may not be directly transferable to other populations. Despite these limitations, we suggest that the underlying relationships observed may still be analytically generalized to other groups facing similar pressures and call for future research to explore these dynamics in more diverse populations, including those with mental health disorders, to enhance our understanding of the complex interplay between social media, body image, and BDD.

Regarding the strength of the study, focusing on medical students allows for a specific demographic that is likely to be aware of mental health issues, potentially leading to more insightful responses. We make sure to avoid suggestive language and any leading questions. In addition, we have delivered the survey anonymously through the web to avoid observers’ bias and to increase confidentiality and privacy. We also ensured that the survey was short to avoid responder bias due to fatigue. Examining BDD concerning social media use is particularly relevant in today’s digital age, where social media can significantly impact self-image and mental health. Furthermore, the questionnaire can yield quantifiable data that can be statistically analyzed, allowing for clear associations and trends to be identified. Respondents may feel more comfortable providing honest answers about sensitive topics like body image and mental health in an anonymous survey than in a face-to-face interview. We recommend a future study that compares between body concern questionnaire (BCQ) and BDDQ in terms of sensitivity and specificity.

## Conclusion

Our study found that BDD is highly prevalent among social media users, especially text-based platforms. The prevalence of BDD among Egyptian medical students is 6.7%, which is higher than worldwide. Interestingly, Egyptian medical students enrolled in our study believe that promoting body positivity, educating users about the risks of body dysmorphia, restricting content that promotes unrealistic body standards, and providing resources and support for those affected, respectively, are the critical measures that social media platforms should take to address body dysmorphia. Furthermore, they strongly agree with initiatives aimed at raising awareness about social media-related body dysmorphia. Most participants with issues with their appearance do not like their faces, their abdomen, and noses. Among those who suffered in school or work, the majority reported bullying and social anxiety. This research is significant in its offering of insight into the traits and social media behaviors of Egyptian medical students, and their possible link to Body Dysmorphic Disorder (BDD). Our results indicate statistically significant differences between the BDD and non-BDD groups about age and governorate, with the BDD group being slightly older overall and having clear-cut distributions by different governorates. In addition, a strong association was found between BDD and economic status and place of residence. While social media usage patterns, including platform usage, time, selfie behavior, filter use, and posting frequency, did not show statistically significant differences between the two groups, the trends observed are worthy of further investigation. The high prevalence of social media usage and selfie retouching among medical students highlights the potential effect of digitally altered images on body image perception. More comprehensive research involving larger samples and more subtle assessments of social media use is needed to further clarify the dynamic interplay among social media, sociocultural factors, and the risk of BDD in this population. Last but not least, the findings of these studies underscore the importance of raising awareness regarding body image issues and enhancing media literacy among Egyptian youth.

## Data Availability

The datasets used and/or analysed during the current study available from the corresponding author on reasonable request.
